# Tris(aceto­nitrile-κ*N*)dichlorido(tri­phenyl­phosphane-κ*P*)ruthenium(II) aceto­nitrile monosolvate

**DOI:** 10.1107/S1600536813014128

**Published:** 2013-05-25

**Authors:** Xiao-Feng Yin, Yi Qin, Hua-Tian Shi, Qun Chen, Qian-Feng Zhang

**Affiliations:** a Department of Applied Chemistry, School of Petrochemical Engineering, Changzhou University, Jiangsu 213164, People’s Republic of China; b Institute of Molecular Engineering and Applied Chemsitry, Anhui University of Technology, Ma’anshan, Anhui 243002, People’s Republic of China

## Abstract

In the title complex, [RuCl_2_(CH_3_CN)_3_(C_18_H_15_P)]·CH_3_CN, the coordination geometry of the Ru^II^ atom is distorted octa­hedral, defined by one P atom from a tri­phenyl­phosphane ligand, three N atoms from three aceto­nitrile ligands and two Cl atoms. The three acetronitile ligands linearly bind to the Ru^II^ atom, with Ru—N—C angles of 172.6 (2), 179.9 (2) and 171.4 (2)°.

## Related literature

For background to ruthenium complexes, see: Caulton (1974 ▶); Gilbert & Wilkinson (1969 ▶); Hallman *et al.* (1970 ▶); Jansen *et al.* (2000 ▶); Stephenson & Wilkinson (1966 ▶); Trost *et al.* (2001 ▶). For related structures, see: Al-Far & Slaughter (2008 ▶); Naskar & Bhattacharjee (2005 ▶).
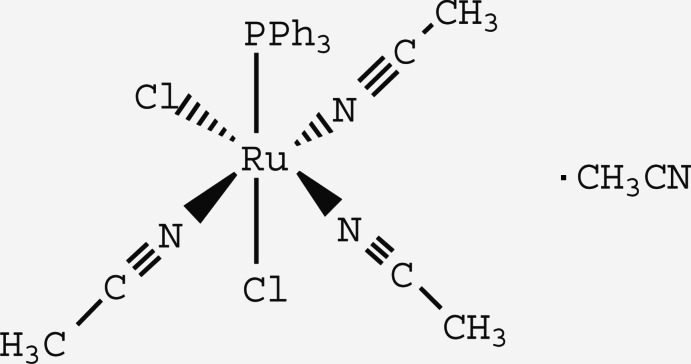


## Experimental

### 

#### Crystal data

[RuCl_2_(C_2_H_3_N)_3_(C_18_H_15_P)]·C_2_H_3_N
*M* *_r_* = 598.46
Monoclinic, 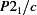

*a* = 15.1133 (13) Å
*b* = 13.9144 (12) Å
*c* = 13.3121 (12) Å
β = 99.275 (2)°
*V* = 2762.8 (4) Å^3^
*Z* = 4
Mo *K*α radiation
μ = 0.84 mm^−1^
*T* = 296 K
0.15 × 0.13 × 0.10 mm

#### Data collection

Bruker APEXII CCD diffractometer
Absorption correction: multi-scan (*SADABS*; Sheldrick, 1996 ▶) *T*_min_ = 0.884, *T*_max_ = 0.921
16271 measured reflections
5403 independent reflections
4544 reflections with *I* > 2σ(*I*)
*R*_int_ = 0.031

#### Refinement

*R*[*F* ^2^ > 2σ(*F* ^2^)] = 0.025
*wR*(*F* ^2^) = 0.063
*S* = 1.04
5403 reflections
311 parameters
H-atom parameters constrained
Δρ_max_ = 0.33 e Å^−3^
Δρ_min_ = −0.33 e Å^−3^

### 

Data collection: *APEX2* (Bruker, 2007 ▶); cell refinement: *SAINT* (Bruker, 2007 ▶); data reduction: *SAINT*; program(s) used to solve structure: *SHELXS97* (Sheldrick, 2008 ▶); program(s) used to refine structure: *SHELXL97* (Sheldrick, 2008 ▶); molecular graphics: *SHELXTL* (Sheldrick, 2008 ▶); software used to prepare material for publication: *SHELXTL*.

## Supplementary Material

Crystal structure: contains datablock(s) I, global. DOI: 10.1107/S1600536813014128/hy2625sup1.cif

Structure factors: contains datablock(s) I. DOI: 10.1107/S1600536813014128/hy2625Isup2.hkl

Additional supplementary materials:  crystallographic information; 3D view; checkCIF report

## Figures and Tables

**Table 1:** Selected bond lengths (Å)

Ru1—N1	2.0218 (19)
Ru1—N2	2.0141 (19)
Ru1—N3	2.0044 (18)
Ru1—P1	2.2754 (6)
Ru1—Cl1	2.4080 (6)
Ru1—Cl2	2.5007 (6)

**Fig. 1.**
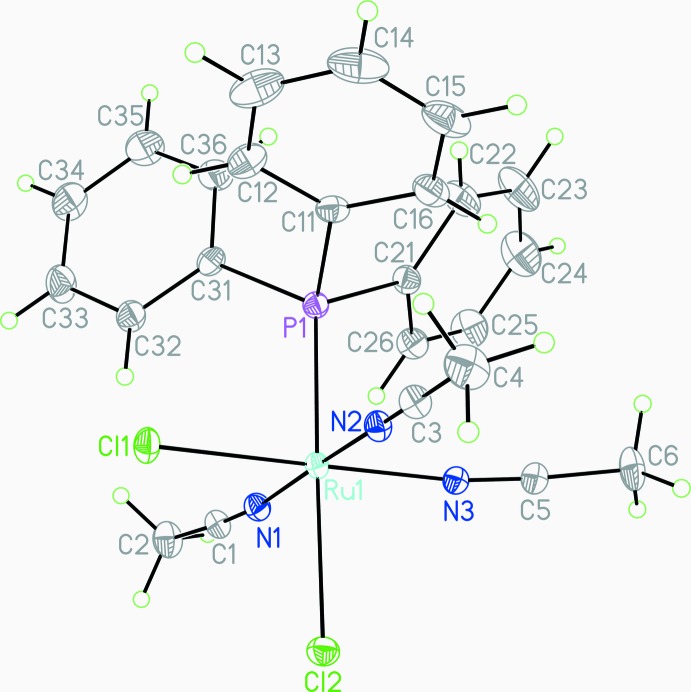
The molecular structure of the title compound. Displacement ellipsoids are drawn at the 50% probability level.

## supplementary crystallographic information

### Comment

The structural and reactivity studies of coordinatively unsaturated ruthenium complexes have received much attention due to their versatile and diverse applications in organic transformation (Jansen *et al.*, 2000; Trost *et al.*, 2001). Since Stephenson & Wilkinson (1966) reported [RuCl_2_(PPh_3_)_3_], it has been found that such five-coordinated ruthenium(II) complex readily loses one triphenylphosphane ligand in solution to give a six-coordinated ruthenium(II) complex with a [Ru(PPh_3_)_2_] moiety (Caulton, 1974). For example, two isomers of [RuCl_2_(CH_3_CN)_2_(PPh_3_)_2_] were easily obtained upon refluxing [RuCl_2_(PPh_3_)_3_] in a mixed solution with acetonitrile as both co-solvent and ligand (Al-Far & Slaughter, 2008; Gilbert & Wilkinson, 1969), which were firstly characterized by infrared spectroscopy (Hallman *et al.*, 1970) and later confirmed by X-ray crystallography (Al-Far & Slaughter, 2008). We found that a similar reaction of [RuCl_2_(PPh_3_)_3_] in the presence of equal equivalent of hydrogen peroxide resulted in loss of two triphenylphosphane ligands and coordination of three acetonitrile ligands. In this paper, we report the synthesis and crystal structure of a new ruthenium(II) complex [RuCl_2_(CH_3_CN)_3_(PPh_3_)].CH_3_CN with a [Ru(PPh_3_)] species.

In the title complex, the coordination geometry of the Ru^II^ atom is a distorted octahedral with one triphenylphosphane, three acetonitriles and two chlorides (Fig. 1). The average Ru—N bond distance value, 2.014 (2) Å, is similar to that found in *cis*-[RuCl_2_(CH_3_CN)_2_(PPh_3_)_2_] [av. 2.010 (2) Å] (Al-Far & Slaughter, 2008). The Ru—N bond distance of the CH_3_CN ligand *trans* to the chloride ligand is not extended (Jansen *et al.*, 2000; Naskar & Bhattacharjee, 2005). Three CH_3_CN ligands are coordinated linearly to the Ru^II^ atom with average Ru—N—C and N—C—C angles of 174.6 (2)° and 177.3 (2)°, respectively. Average Ru—Cl bond distance [2.4542 (6) Å] is as expected (Al-Far & Slaughter, 2008; Jansen *et al.*, 2000). The Ru—P bond length of 2.2754 (6) Å in the title complex is slightly shorter than those of 2.3688 (7) and 2.3887 (7) Å in the complex *cis*-[RuCl_2_(CH_3_CN)_2_(PPh_3_)_2_]. It is interesting to note that the Ru1—Cl2 bond [2.5007 (6) Å] *trans* to the PPh_3_ ligand is ca. 0.1 Å longer than the Ru1—Cl1 bond [2.4080 (6)Å] *trans* to the CH_3_CN ligand. The elongation of the Ru—Cl bond *trans* to the phosphane ligand is probably due to a relatively strong σ back-bonding from phosphrous to ruthenium.

### Experimental

[RuCl_2_(PPh_3_)_3_] (191 mg, 0.2 mmol) was dissolved in a freshly distilled CH_3_CN (10 ml), with stirring at room temperature for 30 min. During this time, the color of the solution was changed from dark brown to orange. Hydrogen peroxide (30%, 6.8 ml, 0.2 mmol) was added to the solution and then the reaction mixture was stirred at reflux for 15 min, developing a yellow. The solvent was evaporated in vacuo and the yellow residue was washed with diethyl ether. Recrystallization from CH_3_CN/Et_2_O afforded yellow crystals of the title complex within five days. Yield: 127 mg, 69% (based on Ru). Analysis, calculated for C_26_H_27_Cl_2_N_4_PRu: C 52.18, H 4.55, N 9.36%; found C 52.13, H 4.51, N 9.39%.

### Refinement

H atoms were placed in geometrically idealized positions and refined as riding atoms, with C—H = 0.93 (aromatic) and 0.96 (methyl) Å and with *U*_iso_(H) = 1.2(1.5 for methyl)*U*_eq_(C).

### Crystal data

**Table:**

[RuCl_2_(C_2_H_3_N)_3_(C_18_H_15_P)]·C_2_H_3_N	*F*(000) = 1216
*M**_r_* = 598.46	*D*_x_ = 1.439 Mg m^−^^3^
Monoclinic, *P*2_1_/*c*	Mo *K*α radiation, λ = 0.71073 Å
Hall symbol: -P 2ybc	Cell parameters from 7259 reflections
*a* = 15.1133 (13) Å	θ = 2.4–29.4°
*b* = 13.9144 (12) Å	µ = 0.84 mm^−^^1^
*c* = 13.3121 (12) Å	*T* = 296 K
β = 99.275 (2)°	Block, yellow
*V* = 2762.8 (4) Å^3^	0.15 × 0.13 × 0.10 mm
*Z* = 4	

### Data collection

**Table:**

Bruker APEXII CCD diffractometer	5403 independent reflections
Radiation source: fine-focus sealed tube	4544 reflections with *I* > 2σ(*I*)
Graphite monochromator	*R*_int_ = 0.031
φ and ω scans	θ_max_ = 26.0°, θ_min_ = 2.0°
Absorption correction: multi-scan (*SADABS*; Sheldrick, 1996)	*h* = −18→16
*T*_min_ = 0.884, *T*_max_ = 0.921	*k* = −17→17
16271 measured reflections	*l* = −16→15

### Refinement

**Table:**

Refinement on *F*^2^	Primary atom site location: structure-invariant direct methods
Least-squares matrix: full	Secondary atom site location: difference Fourier map
*R*[*F*^2^ > 2σ(*F*^2^)] = 0.025	Hydrogen site location: inferred from neighbouring sites
*wR*(*F*^2^) = 0.063	H-atom parameters constrained
*S* = 1.04	*w* = 1/[σ^2^(*F*_o_^2^) + (0.0223*P*)^2^ + 1.1432*P*] where *P* = (*F*_o_^2^ + 2*F*_c_^2^)/3
5403 reflections	(Δ/σ)_max_ = 0.001
311 parameters	Δρ_max_ = 0.33 e Å^−^^3^
0 restraints	Δρ_min_ = −0.33 e Å^−^^3^

### Special details

**Table:**

Geometry. All esds (except the esd in the dihedral angle between two l.s. planes) are estimated using the full covariance matrix. The cell esds are taken into account individually in the estimation of esds in distances, angles and torsion angles; correlations between esds in cell parameters are only used when they are defined by crystal symmetry. An approximate (isotropic) treatment of cell esds is used for estimating esds involving l.s. planes.
Refinement. Refinement of F^2^ against ALL reflections. The weighted R-factor wR and goodness of fit S are based on F^2^, conventional R-factors R are based on F, with F set to zero for negative F^2^. The threshold expression of F^2^ > 2sigma(F^2^) is used only for calculating R-factors(gt) etc. and is not relevant to the choice of reflections for refinement. R-factors based on F^2^ are statistically about twice as large as those based on F, and R- factors based on ALL data will be even larger.

### Fractional atomic coordinates and isotropic or equivalent isotropic displacement parameters (Å^2^)

**Table:**

	*x*	*y*	*z*	*U*_iso_*/*U*_eq_	
Ru1	0.392694 (11)	0.357317 (12)	0.697216 (12)	0.02730 (6)	
Cl1	0.34706 (4)	0.31895 (4)	0.52005 (4)	0.04156 (14)	
Cl2	0.55085 (4)	0.30921 (4)	0.68935 (5)	0.04180 (14)	
P1	0.25036 (4)	0.40373 (4)	0.70896 (4)	0.03124 (13)	
N1	0.42461 (13)	0.49189 (14)	0.65831 (14)	0.0346 (4)	
N2	0.36584 (12)	0.22009 (13)	0.72988 (14)	0.0329 (4)	
N3	0.43047 (12)	0.37894 (12)	0.84685 (14)	0.0309 (4)	
N4	0.0609 (3)	0.6282 (3)	0.1682 (4)	0.1235 (15)	
C1	0.43654 (16)	0.56570 (17)	0.62675 (17)	0.0371 (5)	
C2	0.4461 (2)	0.66014 (18)	0.5837 (2)	0.0536 (7)	
H2A	0.3951	0.6733	0.5325	0.080*	
H2B	0.4997	0.6620	0.5536	0.080*	
H2C	0.4498	0.7077	0.6364	0.080*	
C3	0.35075 (16)	0.14273 (17)	0.74815 (18)	0.0380 (5)	
C4	0.3303 (2)	0.04378 (19)	0.7712 (2)	0.0600 (8)	
H4A	0.3235	0.0388	0.8415	0.090*	
H4B	0.3782	0.0027	0.7584	0.090*	
H4C	0.2755	0.0245	0.7290	0.090*	
C5	0.44268 (16)	0.38452 (16)	0.93285 (18)	0.0361 (5)	
C6	0.4521 (2)	0.3943 (2)	1.04268 (18)	0.0560 (8)	
H6A	0.3952	0.4108	1.0611	0.084*	
H6B	0.4948	0.4439	1.0652	0.084*	
H6C	0.4725	0.3346	1.0743	0.084*	
C7	0.1151 (3)	0.6798 (3)	0.1963 (3)	0.0899 (12)	
C8	0.1863 (4)	0.7457 (3)	0.2315 (4)	0.1292 (19)	
H8A	0.2404	0.7104	0.2547	0.194*	
H8B	0.1707	0.7832	0.2866	0.194*	
H8C	0.1956	0.7875	0.1768	0.194*	
C11	0.17380 (15)	0.30216 (17)	0.70816 (19)	0.0391 (5)	
C12	0.12238 (18)	0.27311 (19)	0.6177 (2)	0.0520 (7)	
H12	0.1214	0.3098	0.5591	0.062*	
C13	0.0722 (2)	0.1892 (2)	0.6144 (3)	0.0728 (10)	
H13	0.0376	0.1699	0.5536	0.087*	
C14	0.0735 (2)	0.1349 (2)	0.7002 (4)	0.0794 (11)	
H14	0.0389	0.0794	0.6978	0.095*	
C15	0.1253 (2)	0.1616 (2)	0.7894 (3)	0.0686 (10)	
H15	0.1267	0.1237	0.8472	0.082*	
C16	0.17586 (18)	0.2451 (2)	0.7941 (2)	0.0516 (7)	
H16	0.2113	0.2629	0.8550	0.062*	
C21	0.23964 (16)	0.47418 (18)	0.82343 (18)	0.0399 (6)	
C22	0.1735 (2)	0.4603 (2)	0.8828 (2)	0.0654 (9)	
H22	0.1323	0.4106	0.8682	0.078*	
C23	0.1694 (3)	0.5217 (3)	0.9650 (3)	0.0909 (13)	
H23	0.1250	0.5127	1.0050	0.109*	
C24	0.2298 (3)	0.5948 (3)	0.9876 (3)	0.0882 (12)	
H24	0.2271	0.6343	1.0434	0.106*	
C25	0.2941 (2)	0.6099 (2)	0.9282 (2)	0.0675 (9)	
H25	0.3346	0.6603	0.9427	0.081*	
C26	0.29900 (18)	0.55016 (19)	0.8468 (2)	0.0483 (6)	
H26	0.3430	0.5609	0.8066	0.058*	
C31	0.19006 (16)	0.48562 (16)	0.61322 (18)	0.0374 (5)	
C32	0.22665 (18)	0.51600 (19)	0.53028 (19)	0.0466 (6)	
H32	0.2819	0.4920	0.5198	0.056*	
C33	0.1818 (2)	0.5820 (2)	0.4623 (2)	0.0635 (8)	
H33	0.2071	0.6015	0.4064	0.076*	
C34	0.1014 (2)	0.6185 (2)	0.4766 (3)	0.0709 (9)	
H34	0.0721	0.6635	0.4314	0.085*	
C35	0.0644 (2)	0.5885 (3)	0.5579 (3)	0.0785 (11)	
H35	0.0090	0.6128	0.5675	0.094*	
C36	0.10779 (19)	0.5228 (2)	0.6261 (2)	0.0609 (8)	
H36	0.0815	0.5032	0.6813	0.073*	

### Atomic displacement parameters (Å^2^)

**Table:**

	*U*^11^	*U*^22^	*U*^33^	*U*^12^	*U*^13^	*U*^23^
Ru1	0.03176 (10)	0.02563 (10)	0.02496 (10)	0.00084 (7)	0.00587 (7)	0.00132 (7)
Cl1	0.0517 (4)	0.0445 (3)	0.0275 (3)	0.0104 (3)	0.0035 (2)	−0.0018 (2)
Cl2	0.0371 (3)	0.0440 (3)	0.0454 (3)	0.0047 (3)	0.0098 (3)	0.0021 (3)
P1	0.0319 (3)	0.0319 (3)	0.0300 (3)	0.0012 (2)	0.0053 (2)	0.0004 (2)
N1	0.0382 (11)	0.0327 (11)	0.0341 (10)	0.0009 (8)	0.0092 (8)	0.0015 (9)
N2	0.0377 (11)	0.0326 (11)	0.0291 (10)	0.0022 (8)	0.0074 (8)	0.0001 (8)
N3	0.0336 (10)	0.0278 (10)	0.0306 (10)	−0.0005 (8)	0.0037 (8)	0.0012 (8)
N4	0.119 (3)	0.112 (3)	0.143 (4)	−0.016 (3)	0.032 (3)	−0.026 (3)
C1	0.0415 (13)	0.0381 (13)	0.0330 (12)	−0.0016 (11)	0.0098 (10)	−0.0002 (11)
C2	0.0728 (19)	0.0377 (14)	0.0508 (16)	−0.0087 (13)	0.0116 (14)	0.0101 (12)
C3	0.0416 (13)	0.0354 (13)	0.0379 (13)	0.0009 (11)	0.0090 (10)	−0.0009 (11)
C4	0.072 (2)	0.0343 (14)	0.076 (2)	−0.0049 (14)	0.0177 (16)	0.0093 (14)
C5	0.0438 (14)	0.0269 (11)	0.0365 (14)	−0.0040 (10)	0.0029 (10)	0.0004 (10)
C6	0.091 (2)	0.0459 (15)	0.0293 (13)	−0.0197 (15)	0.0056 (13)	−0.0005 (12)
C7	0.123 (4)	0.072 (3)	0.076 (3)	0.001 (3)	0.019 (3)	−0.005 (2)
C8	0.182 (5)	0.092 (3)	0.104 (4)	−0.038 (4)	−0.009 (3)	−0.002 (3)
C11	0.0309 (12)	0.0373 (13)	0.0499 (14)	0.0019 (10)	0.0092 (10)	0.0027 (11)
C12	0.0465 (15)	0.0428 (15)	0.0629 (18)	0.0015 (12)	−0.0027 (13)	−0.0038 (13)
C13	0.0510 (18)	0.0516 (18)	0.109 (3)	−0.0075 (15)	−0.0077 (18)	−0.020 (2)
C14	0.055 (2)	0.0468 (18)	0.141 (4)	−0.0130 (16)	0.031 (2)	0.000 (2)
C15	0.060 (2)	0.0530 (18)	0.101 (3)	−0.0012 (15)	0.038 (2)	0.0203 (18)
C16	0.0469 (16)	0.0522 (16)	0.0596 (17)	0.0006 (13)	0.0198 (13)	0.0093 (14)
C21	0.0419 (14)	0.0425 (14)	0.0364 (13)	0.0090 (11)	0.0097 (10)	−0.0020 (11)
C22	0.072 (2)	0.068 (2)	0.0646 (19)	−0.0030 (17)	0.0352 (17)	−0.0113 (16)
C23	0.119 (3)	0.095 (3)	0.075 (2)	−0.002 (3)	0.065 (2)	−0.021 (2)
C24	0.121 (3)	0.081 (3)	0.070 (2)	0.001 (2)	0.039 (2)	−0.033 (2)
C25	0.081 (2)	0.0577 (18)	0.065 (2)	0.0018 (17)	0.0141 (17)	−0.0229 (16)
C26	0.0512 (16)	0.0458 (15)	0.0493 (15)	0.0056 (12)	0.0127 (12)	−0.0085 (12)
C31	0.0378 (13)	0.0335 (12)	0.0387 (13)	0.0056 (10)	−0.0003 (10)	0.0023 (10)
C32	0.0465 (15)	0.0497 (15)	0.0435 (14)	0.0150 (12)	0.0067 (11)	0.0074 (12)
C33	0.073 (2)	0.069 (2)	0.0502 (17)	0.0246 (17)	0.0128 (15)	0.0207 (15)
C34	0.069 (2)	0.072 (2)	0.069 (2)	0.0335 (17)	0.0036 (17)	0.0239 (17)
C35	0.0556 (19)	0.097 (3)	0.084 (2)	0.0408 (19)	0.0133 (17)	0.023 (2)
C36	0.0456 (16)	0.074 (2)	0.0648 (19)	0.0170 (15)	0.0137 (14)	0.0158 (16)

### Geometric parameters (Å, º)

**Table:**

Ru1—N1	2.0218 (19)	C12—C13	1.389 (4)
Ru1—N2	2.0141 (19)	C12—H12	0.9300
Ru1—N3	2.0044 (18)	C13—C14	1.367 (5)
Ru1—P1	2.2754 (6)	C13—H13	0.9300
Ru1—Cl1	2.4080 (6)	C14—C15	1.365 (5)
Ru1—Cl2	2.5007 (6)	C14—H14	0.9300
P1—C11	1.825 (2)	C15—C16	1.386 (4)
P1—C31	1.838 (2)	C15—H15	0.9300
P1—C21	1.841 (2)	C16—H16	0.9300
N1—C1	1.135 (3)	C21—C22	1.384 (3)
N2—C3	1.135 (3)	C21—C26	1.389 (4)
N3—C5	1.133 (3)	C22—C23	1.398 (4)
N4—C7	1.108 (5)	C22—H22	0.9300
C1—C2	1.450 (3)	C23—C24	1.368 (5)
C2—H2A	0.9600	C23—H23	0.9300
C2—H2B	0.9600	C24—C25	1.363 (5)
C2—H2C	0.9600	C24—H24	0.9300
C3—C4	1.455 (3)	C25—C26	1.377 (4)
C4—H4A	0.9600	C25—H25	0.9300
C4—H4B	0.9600	C26—H26	0.9300
C4—H4C	0.9600	C31—C32	1.379 (3)
C5—C6	1.452 (3)	C31—C36	1.383 (4)
C6—H6A	0.9600	C32—C33	1.387 (4)
C6—H6B	0.9600	C32—H32	0.9300
C6—H6C	0.9600	C33—C34	1.358 (4)
C7—C8	1.434 (6)	C33—H33	0.9300
C8—H8A	0.9600	C34—C35	1.361 (5)
C8—H8B	0.9600	C34—H34	0.9300
C8—H8C	0.9600	C35—C36	1.379 (4)
C11—C12	1.384 (4)	C35—H35	0.9300
C11—C16	1.389 (4)	C36—H36	0.9300
			
N3—Ru1—N2	87.87 (7)	C12—C11—P1	119.8 (2)
N3—Ru1—N1	94.25 (7)	C16—C11—P1	120.5 (2)
N2—Ru1—N1	176.40 (7)	C11—C12—C13	120.0 (3)
N3—Ru1—P1	90.58 (5)	C11—C12—H12	120.0
N2—Ru1—P1	91.64 (5)	C13—C12—H12	120.0
N1—Ru1—P1	91.26 (5)	C14—C13—C12	120.3 (3)
N3—Ru1—Cl1	175.81 (5)	C14—C13—H13	119.9
N2—Ru1—Cl1	88.02 (5)	C12—C13—H13	119.9
N1—Ru1—Cl1	89.82 (6)	C15—C14—C13	120.3 (3)
P1—Ru1—Cl1	90.31 (2)	C15—C14—H14	119.8
N3—Ru1—Cl2	87.75 (5)	C13—C14—H14	119.8
N2—Ru1—Cl2	89.00 (5)	C14—C15—C16	120.2 (3)
N1—Ru1—Cl2	88.17 (5)	C14—C15—H15	119.9
P1—Ru1—Cl2	178.19 (2)	C16—C15—H15	119.9
Cl1—Ru1—Cl2	91.40 (2)	C15—C16—C11	120.2 (3)
C11—P1—C31	103.47 (11)	C15—C16—H16	119.9
C11—P1—C21	106.07 (11)	C11—C16—H16	119.9
C31—P1—C21	98.27 (11)	C22—C21—C26	118.6 (2)
C11—P1—Ru1	112.66 (8)	C22—C21—P1	124.5 (2)
C31—P1—Ru1	119.79 (8)	C26—C21—P1	116.80 (18)
C21—P1—Ru1	114.73 (8)	C21—C22—C23	119.3 (3)
C1—N1—Ru1	172.6 (2)	C21—C22—H22	120.4
C3—N2—Ru1	179.9 (2)	C23—C22—H22	120.4
C5—N3—Ru1	171.40 (18)	C24—C23—C22	120.9 (3)
N1—C1—C2	176.5 (3)	C24—C23—H23	119.5
C1—C2—H2A	109.5	C22—C23—H23	119.5
C1—C2—H2B	109.5	C25—C24—C23	120.0 (3)
H2A—C2—H2B	109.5	C25—C24—H24	120.0
C1—C2—H2C	109.5	C23—C24—H24	120.0
H2A—C2—H2C	109.5	C24—C25—C26	119.8 (3)
H2B—C2—H2C	109.5	C24—C25—H25	120.1
N2—C3—C4	179.3 (3)	C26—C25—H25	120.1
C3—C4—H4A	109.5	C25—C26—C21	121.4 (3)
C3—C4—H4B	109.5	C25—C26—H26	119.3
H4A—C4—H4B	109.5	C21—C26—H26	119.3
C3—C4—H4C	109.5	C32—C31—C36	118.0 (2)
H4A—C4—H4C	109.5	C32—C31—P1	121.83 (18)
H4B—C4—H4C	109.5	C36—C31—P1	120.1 (2)
N3—C5—C6	176.0 (3)	C31—C32—C33	120.6 (2)
C5—C6—H6A	109.5	C31—C32—H32	119.7
C5—C6—H6B	109.5	C33—C32—H32	119.7
H6A—C6—H6B	109.5	C34—C33—C32	120.7 (3)
C5—C6—H6C	109.5	C34—C33—H33	119.7
H6A—C6—H6C	109.5	C32—C33—H33	119.7
H6B—C6—H6C	109.5	C33—C34—C35	119.2 (3)
N4—C7—C8	178.9 (6)	C33—C34—H34	120.4
C7—C8—H8A	109.5	C35—C34—H34	120.4
C7—C8—H8B	109.5	C34—C35—C36	120.9 (3)
H8A—C8—H8B	109.5	C34—C35—H35	119.5
C7—C8—H8C	109.5	C36—C35—H35	119.5
H8A—C8—H8C	109.5	C35—C36—C31	120.5 (3)
H8B—C8—H8C	109.5	C35—C36—H36	119.7
C12—C11—C16	119.0 (2)	C31—C36—H36	119.7
